# User Behavior of a Publicly Available, Free-to-Use, Self-guided mHealth App for Depression: Observational Study in a Global Sample

**DOI:** 10.2196/35538

**Published:** 2022-10-25

**Authors:** Langting Su, Page Lyn Anderson

**Affiliations:** 1 Department of Psychology Georgia State University Atlanta, GA United States; 2 Inquiry Health, LLC Sheridan, WY United States

**Keywords:** mHealth, depression, smartphone, mobile app, digital health, global mental health, MoodTools, mobile health, mental health, mobile phone, low- and middle-income countries

## Abstract

**Background:**

Reducing the burden of depression is a global health concern. Self-guided mobile health (mHealth) apps are one approach to address this problem. However, there is little research on self-guided mHealth apps in a global sample or on how they are used in the real world. These gaps in our knowledge must be addressed to bring the promise of mHealth apps for reducing the global burden of depression closer to reality.

**Objective:**

The purpose of this study is to examine the naturalistic user behavior of MoodTools, a publicly available, free-to-use, self-guided mHealth app designed to improve symptoms of depression, in a global community sample.

**Methods:**

Mobile analytics data were collected from all unique downloads of the Android version of MoodTools between March 1, 2016, and February 28, 2018. Due to the deidentification and data aggregation process, no demographic or personal identifying information was tied to individual user data. All information was stored in aggregated, anonymized data files on Google Analytics’ storage database. Google’s software development kit was used to securely capture data about the number of downloads, location of downloads, number of app sessions, frequency and duration of app sessions, time between app sessions, and user retention, allowing for examination of which app’s tools were viewed and for how long, including *Information* (psychoeducation), *Test* (self-monitoring using the Patient Health Questionnaire [PHQ-9]), *Thought Diary* (targeting negative cognitions), *Activities* (behavioral activation), *Videos* (curated from YouTube), and *Safety Plan* (safety plan development and links to quickly access crisis management resources).

**Results:**

MoodTools was used by 158,930 people from 198 countries, including countries where English was not the primary language and in low- and middle-income countries. After the initial download, 51.14% (n=81,277) of users returned to the app after the initial download, and retention rates decreased with each subsequent app session. The typical person used the app for 3 sessions for a total of 12 minutes over 90 days. The most frequently visited tools were *Test* and *Thought Diary* (n=393,549, 24.32%). On average, users completed and reviewed the results of the PHQ-9 for 49 seconds and 53 seconds, respectively, and spent 3 minutes and 5 seconds on *Thought Diary*.

**Conclusions:**

Self-guided mHealth apps could be one approach (among the many needed) to reduce the burden of depression. Observational data collected in this study show a global interest in MoodTools, including in low- and middle-income countries and countries where English is not the primary language. Future research is needed to determine whether people who use self-guided apps experience improvement in depressive symptoms, and if so, what “dosage” provides a meaningful benefit.

## Introduction

Mobile health (mHealth) is any medical or health practice supported by mobile devices such as smartphones [1]. An estimated 5.2 billion people have mobile devices [2], so mHealth interventions have the potential to reach almost two-thirds of the world’s population. Depression is a leading cause of global disability [3] that is associated not only with personal suffering but also unemployment, poor physical health, poor social function, and suicide [4,5]. Pharmacotherapy and psychotherapy improve depressive symptoms, but there are widespread shortages of trained mental health professionals to deliver these therapies that are not expected to improve in the near future [6,7], which limits the reach of these efficacious interventions. Self-guided internet-based interventions can be accessed through the web and used without support from mental health professionals to deliver cost-effective behavioral health services worldwide [8,9]. Specifically, internet-based interventions for depression began gaining traction in the 2000s with Beating the Blues [10,11] and MoodGYM [12], both of which use a fixed sequence navigation format, in which each session is built on the preceding one until the intervention is completed. Although internet-based interventions are efficacious for the treatment of depression [13], nearly half of the participants in clinical trials do not complete a full course of these interventions [14], and the dissemination and implementation of internet-based interventions for depression remain limited [15,16]. With the advent of smartphones in the late 2000s, mobile technology and interventions delivered via downloadable apps became more accessible. Smartphone-based interventions for depression have been shown to improve depressive symptoms [17] and have the potential to address depression worldwide, as the rate of smartphone adoption globally is 65% and is on the rise in countries with emerging economies [18,19].

There is a gap between how people use mHealth interventions in the real world and how researchers evaluate them. Although randomized controlled and feasibility trials find that mobile and web-based interventions are efficacious, the findings do not hold in practical settings [16,20,21]. Many studies use research-only versions of interventions that are not available to the public afterward. Out of 18 apps that were recently reviewed in a meta-analysis of randomized clinical trials evaluating smartphone interventions for depression and anxiety, only 5 are currently available for public download [17]. Conversely, some of the more popular behavioral health apps have tens of millions of downloads on the app marketplace [22], but most are unevaluated [23]. The research gap on mHealth interventions and real-world usage must be addressed to bring the promise of mHealth for reducing the global burden of depression closer to reality.

There is very little ecologically valid research on the use of publicly available self-guided mHealth interventions for mental health [24]. To date, researchers have described the naturalistic behavior of users of apps targeting psychological disorders in only two studies [25,26]. IntelliCare is a suite of 13 smartphone apps designed to improve symptoms of depression and anxiety. Each self-guided app is accessed via a central hub and targets a single function (eg, cultivating gratitude). PTSD Coach is a free, publicly available self-guided app created by the United States Department of Veterans Affairs and the Department of Defense for managing acute distress related to posttraumatic stress disorder [27]. Return-visit users of PTSD Coach reported higher momentary distress levels compared to first-time users, suggesting that the app is being used in moments of need.

There are very few studies on self-guided apps for mental health and well-being within a global sample. PTSD Coach was downloaded in 86 countries, with non-US downloads making up 12% of total downloads [25]. A preliminary study of Wysa, a self-guided artificial intelligence–based chatbot app designed to promote mental well-being using a text-based conversational interface, reported that among the global community of users sampled, participants in the study downloaded the app in the United States, Europe, and Asia [28]. A randomized controlled trial of Headspace, a self-guided app that teaches mindfulness practice through guided meditations, found that its participants represented 11 countries [29]. These studies suggest that there is a global interest in using self-guided apps to improve mental health.

This study aims to describe how MoodTools, a publicly available, free-to-use, self-guided mental health app for depressive symptoms, is used “in the wild” among a global sample.

**Figure 1:**
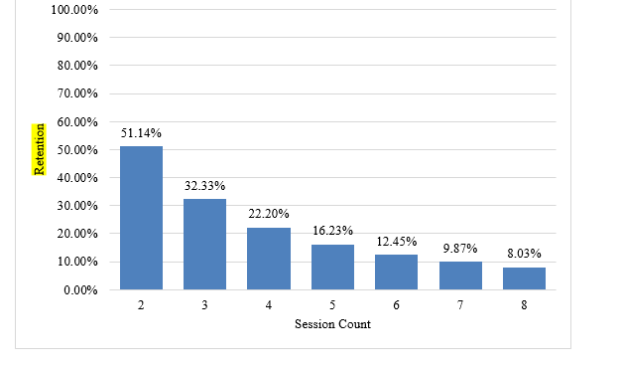
User retention across sessions.

**Figure 2:**
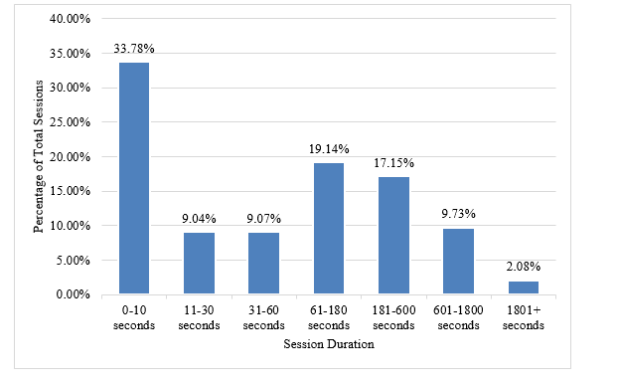
Duration of MoodTools sessions.

**Figure 3:**
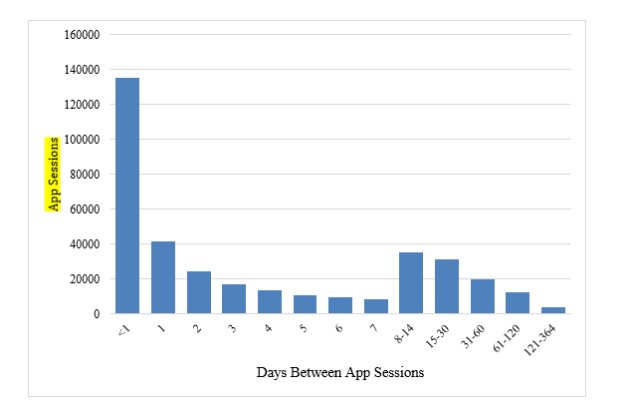
The amount of time between downloading MoodTools and returning to the app.

## Methods

### Overview of MoodTools

MoodTools was published on Google Play for Android devices in June 2014 and on Apple App Store for iOS devices in 2015. Since its release in 2014, it has been downloaded on iOS and Android devices over 500,000 times. MoodTools is a fully automated, self-guided smartphone app for iOS (ie, iPhone and iPad) and Android devices. All content is self-contained, with the exception of external links that take the users out of the app; there is no human interaction. The app is exclusively in English. MoodTools contains 6 features called tools. The *Information* tool contains psychoeducation about depression. The *Test* tool contains the mobile form of the Patient Health Questionnaire (PHQ-9), a 9-item depression screening questionnaire that has been validated in the paper form [30] and in the mobile form [31]. If a user responds with “more than half the days” or “nearly every day” to item 9 of the PHQ-9 (ie, “Over the last 2 weeks, how often have you been bothered by thoughts that you would be better off dead or of hurting yourself in some way”), the results page displays a link to the country-specific suicide hotline if it was available in that country and external resources for managing suicidal thoughts. The *Thought Diary* tool features a diary entry derived from thought records [32] for the practice of thought restructuring. The *Activities* tool, based on behavioral activation therapy, prompts users to engage in activities to improve their mood. The activities are customizable, and the history page allows users to see which activities provide the most significant boost in subjective mood. The *Videos* tool contains a curated list of YouTube videos such as TED Talks, guided meditations, and soothing sounds for mindfulness. The *Safety Plan* tool gives users information on coping with suicidal thoughts, allows users to fill out a safety plan, and provides quick access to local urgent care, emergency departments, and national crisis hotlines.

### Data Extraction

Data were derived from mobile analytics data from all unique downloads of the Android version of MoodTools between March 1, 2016, and February 28, 2018. Mobile analytics data were not collected from the iOS app during this study period. Due to the deidentification and data aggregation process, no demographic or personal identifying information was tied to individual user data. All measures were stored in aggregated, anonymized data files on Google Analytics’ storage database.

The Google Analytics software development kit (SDK) was integrated into the Android app in March 2016. We used the SDK to securely capture aggregate usage data and retention information from March 2016 to 2018 (the maximum amount of aggregate data that can be analyzed at a time). The following information was collected:

- Location: The country from which the app was installed on a user’s Android device.
- Number of downloads: The number of individual users who downloaded the app.
- App session: A single period of user interaction within the app. Activity that occurs within 30 minutes of each other is counted as part of the same app session. If there is no activity for 30 minutes, future activity is attributed to a new session.
- Session frequency: The number of app sessions during the data collection.
- Session duration: The number of minutes the app is open or used during an app session.
- Session recency: The amount of elapsed time since a user’s last app session.
- Total duration in app: The total amount of time spent across app sessions for an individual user.
- Tools visited: The number of times a user opens the home page for each of the 6 tools within the app.

### Ethics Approval

This study was approved by the Institutional Review Board at Georgia State University as Designation for Not Human Subjects Research (H21568).

## Results

### Downloads

Between March 1, 2016, and February 28, 2018, MoodTools on the Android platform was used by 158,930 people from 198 countries. Data were collected on the percentage of users by continent, subcontinent, and country (see Multimedia Appendix 1). The app was downloaded across the Americas (50.46%), Europe (26.46%), Asia (15.48%), Oceania (4.82%), and Africa (2.61%). When categorized by subcontinents, more than half of all users were from Northern America and Northern Europe (46.537% and 13.324%, respectively). Countries with the highest percentage of users included the United States (40.83%), the United Kingdom (10.64%), India (8.47%), Canada (5.60%), and Australia (4.02%). There were downloads in low- and middle-income countries, including India (8.47%), Brazil (1.11%), and South Africa (1.01%). Users whose location could not be determined by Google Analytics were identified as “Not Set” (0.18%).

### Retention

Retention was defined as the percentage of users that return to the app at any point after their initial app session. As shown in Table 1, 51.14% (n=81,277) of MoodTools users returned to the app for a second app session. The retention rate declined with each subsequent app session: session 3 (n=51,382, 32.33%), session 4 (n=35,282, 22.2%), session 5 (n=25,794, 16.23%), session 6 (n=19,787, 12.45%), session 7 (n=15,686, 9.87%), and session 8 (n=12,762, 8.03%).

**Table 1:** User retention across sessions.

	User retention, n (%)
2 sessions	81,277 (51.14)
3 sessions	51,382 (32.33)
4 sessions	35,282 (22.2)
5 sessions	25,794 (16.23)
6 sessions	19,787 (12.45)
7 sessions	15,686 (9.87)
8 sessions	12,762 (8.03)

### Time Spent

Users spent 4 minutes, on average, on each session. About one-third of sessions lasted between 0 and 10 seconds, one-third lasted between 11 seconds and 3 minutes, and the remainder lasted more than 3 minutes (Table 2). Over one-third of all app sessions were initiated within the same day of download, and about 1% of sessions occurred following a 3-month to 1-year period of inactivity (Table 3). After downloading MoodTools, the typical person used the app for 3 sessions for a total of 12 minutes over 90 days.

**Table 2:** Duration of MoodTools sessions.

Session duration	Sessions, %
0-10 s	33.78
11-30 s	9.04
31-60 s	9.07
61-180 s	19.14
180-600 s	17.15
601-1800 s	9.73
≥1801 s	2.08

**Table 3:** The amount of time between MoodTools app sessions.

Days between app sessions	App sessions, n (%)
<1 day	134,987 (37.16)
1 day	41,653 (11.47)
2 days	24,187 (6.66)
3 days	17,256 (4.74%)
4 days	13,365 (3.68%)
5 days	10,972 (3.02)
6 days	9820 (2.70)
7 days	8184 (2.25)
8-14 days	35,334 (9.73)
15-30 days	31,306 (8.62)
31-60 days	20,017 (5.51)
61-120 days	12,174 (3.35)
121-364 days	4041 (1.11)

### Tools Visited

We examined how often users visited each of the 6 tools (Thought Diary, Test, Information, Activities, Videos, and Safety Plan). Visiting a tool was operationalized as opening the home page screen for that tool. The Thought Diary tool and Test tool were tied for the most frequently visited tools, each making up 24.32% (n=393,487) of all home page screens viewed across all app sessions for all users (N=1,618,277 total screen views; Table 4). The Information tool (ie, psychoeducation about depression) was the least frequently visited tool (n=1,246,667, 7.7%). Users spent an average of 3 minutes and 5 seconds (185 seconds) on the Thought Diary’s entry screen, which allows users to complete a digital thought record. Users spent an average of 49 seconds completing the PHQ-9 screening questionnaire and an average of 53 seconds on the screen that displayed the results of the PHQ-9.

**Table 4:** Home page views by tool.

Tool name	Total screen views (N=1,618,277), n (%)	Average screen views per app session	Average time on home page screen(s)
Thought Diary	393,549 (24.32)	2.24	12.25
Test	393,487 (24.32)	2.00	5.71
Activities	331,961 (20.51)	2.35	10.08
Safety Plan	236,449 (14.61)	2.32	14.20
Videos	138,164 (8.54)	1.40	5.76
Information	124,667 (7.70)	1.23	11.34

## Discussion

### Principal Results

MoodTools was downloaded in 198 countries, suggesting that there is global interest in a free-to-use self-guided smartphone app for depression. It is worth noting that the developers of MoodTools had done no marketing campaigns and that, even though the app is presented exclusively in English, there have been downloads in countries where English is not the primary language. Users from low- and middle-income countries downloaded the app as well. Despite widespread interest, self-guided mental health apps will not make an impact on the global burden of depression if they are not effective. A review of evidence-based apps for anxiety and depression showed that a large majority (74%) were free to download, but only 3% had research to justify claims of effectiveness [33]. Efficacy studies remain rare in the ever-changing landscape of publicly available mHealth apps. The efficacy and effectiveness of MoodTools (and other mHealth apps for depression) is clearly an important area for future research.

A key challenge for mHealth interventions for depression is to engage and retain users, given the low motivation and behavioral avoidance associated with the condition. Just over half of MoodTools users (n=81,277, 51.14%) returned to the app after the initial download, which is comparable to IntelliCare (about 50%) and PTSD Coach (61.1%) [25,26]. These return rates are similar to the pooled dropout rate (47.8%) reported in a meta-analysis of randomized clinical trials of smartphone apps for depressive symptoms [34]. Return rates of users “in the wild” and dropout rates from efficacy trials for smartphone apps is not, however, an apples-to-apples comparison. Dropout in clinical trials is defined as the incompletion of end-of-intervention assessments, which are generally collected outside of the smartphone app of interest, whereas retention in studies of naturalistic user behavior is typically defined as using the app. Users who download and open a free mHealth app for depression on the app marketplace may have different levels of interest and intent compared to participants who agree to use an mHealth app as part of a clinical trial. Given our limited knowledge of the retention rates of self-guided mental health apps in the real world, it is difficult to determine what is considered “standard” retention within this category of mHealth interventions. Future clinical trials could measure engagement-related dropout as well as assessment-related dropout, and real-world mHealth apps could adopt a similar standardized assessment structure to better compare retention and engagement across these methodologies.

It is important to determine how much app use is needed for meaningful improvement in symptoms. Research indicates that there is a relationship between app use and clinically meaningful benefit [35-37], but a minimum “dose” has not been identified. Around one-third of all MoodTools sessions lasted 10 seconds or less. These “touch-and-go” app sessions are likely too short to be meaningful. The high frequency of these app sessions creates noise in the data and highlights the importance of understanding the conditions under which users stay engaged with an app session long enough to have a meaningful interaction with it.

The dose achieved by typical MoodTools users was 3 app sessions (averaging 4 minutes per session), for a total of 12 minutes over 90 days, which can be compared to PTSD Coach users, who initiated 6.3 app sessions (averaging 47 seconds per session) for a total of 5 minutes before discontinuing its use [25]. In psychotherapy, which is typically delivered weekly in 50-minute sessions, about 50% and 75% of patients improve after 8 and 26 sessions, respectively [38,39]. In this context, it is difficult to imagine that typical MoodTools users achieve a dose that would lead to meaningful improvement in depressive symptoms, though this is an empirical question that remains to be tested. There were, however, a small proportion of users who returned to the app for 9 sessions or more, and some who used the app >200 times.

Unlike mental health professionals, everyday users of mental health apps are not trained to use the science of psychopathology to relieve symptoms. It is critical to identify what users of self-guided mental health apps naturally gravitate to, and the results are encouraging. The most frequently visited tools for MoodTools users were Thought Diary (thought record) and Test (mood self-monitoring). These results are consistent with research on naturalistic user behavior of other smartphone apps, despite differences in layout and psychological problems targeted. The most-visited areas of PTSD Coach were Self-Assessment (symptom tracking) and Manage Symptoms (coping skills) [25], and the most downloaded apps from IntelliCare’s suite were Thought Challenger (thought restructuring) and Worry Knot (worry management) [26]. These studies show that the users of publicly available, self-guided mental health apps most often visit cognitive restructuring and symptom tracking tools. These tools may be more popular because they engage users through active participation (eg, recording symptoms) as compared to tools that are more passive (eg, reading information about the symptoms of depression). Negative cognitions are a core feature of depression, so it is encouraging that users of MoodTools most often visit the Thought Diary tool.

### Limitations

This study of naturalistic user behavior has several notable limitations. First, this study only included users from the Android platform of MoodTools and differences between those who use Android and iOS devices may influence user behavior. iOS users are more likely than Android users to be female, more educated, belong to a higher income group, and have more technical knowledge [40], and there are differences in user reviews between iOS and Android platforms [25]. Second, the deidentified, aggregate nature of data obtained through Google Analytics limits the types of analyses that can be conducted. For example, data on individual user characteristics (eg, gender, age, socioeconomic status) were not collected for this cohort.

### Future Directions

The number of areas for future research on self-guided mHealth apps for reducing depression is seemingly infinite. It is critical to improve our understanding of what happens immediately following the download of the app. Consistent with research on other apps, half of all MoodTools users did not return to the app after their first app session. Research is needed to understand how to maintain users’ engagement from the very first app session and to identify users most at risk for discontinuing use. Additionally, it may be helpful to sort users into low- and high-use comparison groups, as was done in the Wysa study [28], to examine how usage and retention patterns may differ across groups. Virtually nothing is known about how individual user characteristics (eg, gender, age, socioeconomic status) impact engagement or retention. The use of app-based reminders or notifications, as well as incentive structures such as gamification, should be examined to see how these app features impact user engagement. Understanding how a mental health app’s content, approachability, and style affects user engagement is another critical next step. The user version of the Mobile Application Rating Scale (uMARS) [41]—the only self-report scale that is developed for use by the general public [42]—can provide subjective data on user perceptions of a mental health app.

### Conclusions

The scope and impact of depression worldwide is breathtaking. Results show a global interest in a publicly available, free-to-use mHealth app designed to improve depressive symptoms, including in low- and middle-income countries and in countries where English is not the primary language. About half of MoodTools users returned to the app after their initial app session. About one-third of all sessions lasted between 0 and 10 seconds, one-third lasted between 11 seconds and 3 minutes, and the remaining third lasted 3 minutes or longer. The average MoodTools user used the app for 3 sessions for a total of 12 minutes over 90 days. Users tend to spend most time using tools designed for self-monitoring of symptoms and for targeting a core mechanism of depressive psychopathology and negative cognitions. Observational data from this study show that self-guided mental health apps could be one among the many approaches needed to reduce the global burden of depression; however, research is needed to determine whether app engagement can lead to symptom improvement.

## Appendix

Multimedia Appendix 1 MoodTools users by continent, subcontinent, and country.

## Abbreviations

mHealth: mobile health
PHQ-9: Patient Health Questionnaire
SDK: software development kit
uMARS: user version of the Mobile Application Rating Scale
