# Supplementary material for: User Behavior of a Publicly Available, Free-to-Use, Self-guided mHealth App for Depression: Observational Study in a Global Sample
Source: JMIR Form Res. 2022 Oct 25;6(10):e35538. doi: 10.2196/35538 (PMC9644247; doi:10.2196/35538)
Supplement: Multimedia Appendix 1 [file formative_v6i10e35538_app1.docx]

Multimedia Appendix 1

MoodTools users by continent, subcontinent, and country.

| **Continent** | **Users (%)** |
| --- | --- |
| Americas | 50.457 |
| Europe | 26.459 |
| Asia | 15.477 |
| Oceania | 4.823 |
| Africa | 2.608 |
| Not Set | 0.177 |

| **Subcontinent** | **Users (%)** |
| --- | --- |
| Northern America | 46.537 |
| Northern Europe | 13.324 |
| Southern Asia | 9.709 |
| Western Europe | 7.169 |
| Australasia | 4.782 |
| Southeast Asia | 3.820 |
| Southern Europe | 3.370 |
| Eastern Europe | 2.760 |
| South America | 2.444 |
| Western Asia | 1.371 |
| Southern Africa | 1.123 |
| Northern Africa | 0.952 |
| Central America | 0.923 |
| Eastern Asia | 0.615 |
| Caribbean | 0.341 |
| Eastern Africa | 0.302 |
| Western Africa | 0.208 |
| Central Asia | 0.030 |
| Middle Africa | 0.016 |
| Melanesia | 0.015 |
| Micronesian Region | 0.012 |
| Polynesia | 0.002 |
| Not Set | 0.176 |

| Country | Users (%) |
| --- | --- |
| United States | 40.8286 |
| United Kingdom | 10.6379 |
| India | 8.4695 |
| Canada | 5.5900 |
| Australia | 4.0220 |
| Germany | 3.8305 |
| Philippines | 1.8301 |
| Brazil | 1.1133 |
| South Africa | 1.0749 |
| Netherlands | 1.0166 |
| Spain | 0.9107 |
| France | 0.9101 |
| New Zealand | 0.7521 |
| Mexico | 0.7323 |
| Poland | 0.7143 |
| Italy | 0.7081 |
| Ireland | 0.7069 |
| Indonesia | 0.6771 |
| Malaysia | 0.6201 |
| Sweden | 0.5978 |
| Pakistan | 0.5941 |
| Russia | 0.5904 |
| Romania | 0.5520 |
| Argentina | 0.5291 |
| Egypt | 0.5148 |
| Switzerland | 0.4944 |
| Austria | 0.4702 |
| Singapore | 0.4622 |
| Portugal | 0.4306 |
| Israel | 0.4101 |
| Greece | 0.3946 |
| Belgium | 0.3847 |
| Finland | 0.3668 |
| Norway | 0.3333 |
| Denmark | 0.3240 |
| Bangladesh | 0.3017 |
| Serbia | 0.2887 |
| United Arab Emirates | 0.2652 |
| Colombia | 0.2546 |
| Croatia | 0.2497 |
| Czechia | 0.2478 |
| Saudi Arabia | 0.1970 |
| Chile | 0.1927 |
| Morocco | 0.1821 |
| Hungary | 0.1716 |
| South Korea | 0.1648 |
| Slovenia | 0.1642 |
| Ukraine | 0.1549 |
| Japan | 0.1499 |
| Hong Kong | 0.1425 |
| Iran | 0.1413 |
| Kenya | 0.1406 |
| Slovakia | 0.1351 |
| Thailand | 0.1351 |
| Bulgaria | 0.1332 |
| Lithuania | 0.1301 |
| Peru | 0.1301 |
| Algeria | 0.1202 |
| Nigeria | 0.1196 |
| Tunisia | 0.1189 |
| China | 0.1128 |
| Estonia | 0.1128 |
| Nepal | 0.1109 |
| Lebanon | 0.0966 |
| Vietnam | 0.0960 |
| Trinidad & Tobago | 0.0892 |
| Bosnia & Herzegovina | 0.0855 |
| Sri Lanka | 0.0799 |
| Iceland | 0.0787 |
| Jamaica | 0.0774 |
| Jordan | 0.0725 |
| Ghana | 0.0719 |
| Latvia | 0.0700 |
| Puerto Rico | 0.0694 |
| Venezuela | 0.0688 |
| North Macedonia | 0.0632 |
| Costa Rica | 0.0607 |
| Ecuador | 0.0595 |
| Taiwan | 0.0576 |
| Mauritius | 0.0496 |
| Kuwait | 0.0483 |
| Dominican Republic | 0.0477 |
| Malta | 0.0471 |
| Cyprus | 0.0458 |
| Belarus | 0.0446 |
| Qatar | 0.0434 |
| Uruguay | 0.0434 |
| Turkey | 0.0403 |
| Guatemala | 0.0353 |
| Oman | 0.0347 |
| Bahrain | 0.0335 |
| Panama | 0.0335 |
| Albania | 0.0328 |
| Bolivia | 0.0316 |
| Iraq | 0.0291 |
| Namibia | 0.0291 |
| Luxembourg | 0.0279 |
| Tanzania | 0.0279 |
| Paraguay | 0.0229 |
| Cambodia | 0.0204 |
| Zimbabwe | 0.0204 |
| Botswana | 0.0186 |
| Moldova | 0.0186 |
| Uganda | 0.0180 |
| El Salvador | 0.0173 |
| Honduras | 0.0167 |
| Azerbaijan | 0.0161 |
| Kazakhstan | 0.0161 |
| Myanmar (Burma) | 0.0155 |
| Syria | 0.0155 |
| Bahamas | 0.0149 |
| Belize | 0.0149 |
| Yemen | 0.0142 |
| Zambia | 0.0142 |
| Ethiopia | 0.0136 |
| Kosovo | 0.0124 |
| Nicaragua | 0.0112 |
| Fiji | 0.0105 |
| Guam | 0.0105 |
| Maldives | 0.0105 |
| Cameroon | 0.0099 |
| Sudan | 0.0099 |
| Côte d’Ivoire | 0.0093 |
| Armenia | 0.0087 |
| Georgia | 0.0081 |
| Guernsey | 0.0081 |
| Aruba | 0.0056 |
| Jersey | 0.0056 |
| Kyrgyzstan | 0.0050 |
| Mozambique | 0.0050 |
| Palestine | 0.0050 |
| Rwanda | 0.0050 |
| Afghanistan | 0.0043 |
| Uzbekistan | 0.0043 |
| Antigua & Barbuda | 0.0037 |
| Barbados | 0.0037 |
| Brunei | 0.0037 |
| Cayman Islands | 0.0037 |
| Libya | 0.0037 |
| Montenegro | 0.0037 |
| Angola | 0.0031 |
| Bermuda | 0.0031 |
| Cuba | 0.0031 |
| Curaçao | 0.0031 |
| Haiti | 0.0031 |
| U.S. Virgin Islands | 0.0031 |
| St. Lucia | 0.0025 |
| Northern Mariana Islands | 0.0025 |
| Papua New Guinea | 0.0025 |
| Senegal | 0.0025 |
| Andorra | 0.0019 |
| Eritrea | 0.0019 |
| Gabon | 0.0019 |
| Guadeloupe | 0.0019 |
| Martinique | 0.0019 |
| Malawi | 0.0019 |
| Tajikistan | 0.0019 |
| Turkmenistan | 0.0019 |
| St. Vincent & Grenadines | 0.0019 |
| Vanuatu | 0.0019 |
| Burkina Faso | 0.0012 |
| Caribbean Netherlands | 0.0012 |
| Bhutan | 0.0012 |
| Dominica | 0.0012 |
| Faroe Islands | 0.0012 |
| Gibraltar | 0.0012 |
| Guyana | 0.0012 |
| St. Kitts & Nevis | 0.0012 |
| Laos | 0.0012 |
| Madagascar | 0.0012 |
| Mongolia | 0.0012 |
| Réunion | 0.0012 |
| Svalbard & Jan Mayen | 0.0012 |
| Somalia | 0.0012 |
| Suriname | 0.0012 |
| Mayotte | 0.0012 |
| Anguilla | <0.001 |
| American Samoa | <0.001 |
| Burundi | <0.001 |
| Benin | <0.001 |
| Congo - Kinshasa | <0.001 |
| Cook Islands | <0.001 |
| Cape Verde | <0.001 |
| Djibouti | <0.001 |
| French Guiana | <0.001 |
| Greenland | <0.001 |
| Gambia | <0.001 |
| Guinea | <0.001 |
| Liechtenstein | <0.001 |
| Macao | <0.001 |
| Montserrat | <0.001 |
| Niger | <0.001 |
